# High Biofilm-Forming Multidrug-Resistant *Salmonella* Infantis Strains from the Poultry Production Chain

**DOI:** 10.3390/antibiotics13070595

**Published:** 2024-06-27

**Authors:** Laura Musa, Valeria Toppi, Valentina Stefanetti, Noah Spata, Maria Cristina Rapi, Guido Grilli, Maria Filippa Addis, Giacomo Di Giacinto, Maria Pia Franciosini, Patrizia Casagrande Proietti

**Affiliations:** 1 Department of Veterinary Medicine and Animal Sciences, University of Milan, 26900 Lodi, Italy; laura.musa@unimi.it (L.M.); maria.rapi@unimi.it (M.C.R.); guido.grilli@unimi.it (G.G.); filippa.addis@unimi.it (M.F.A.); 2 Department of Veterinary Medicine, University of Perugia, 06126 Perugia, Italy; valeria.toppi@dottorandi.unipg.it (V.T.); valentina.stefanetti@collaboratori.unipg.it (V.S.); noah.spata@studenti.unipg.it (N.S.); maria.franciosini@unipg.it (M.P.F.); 3 Department of Human Science and Promotion of Quality Life, San Raffaele Telematic University, 00166 Rome, Italy; 4 Laboratorio di Malattie Infettive degli Animali (MiLab), University of Milan, 26900 Lodi, Italy; 5 Department of Veterinary Sciences, University of Turin, 10095 Turin, Italy; giacomo.digiacinto@unito.it

**Keywords:** biofilm formation, rdar morphotype, multidrug-resistant (MDR), *Salmonella* virulence factors, *Salmonella* environmental persistence, biosecurity, disinfection

## Abstract

The ability of *Salmonella* species to adhere to surfaces and form biofilms, leading to persistent environmental reservoirs, might represent a direct link between environmental contamination and food processing contamination. The purpose of this study was to investigate the biofilm-forming ability of 80 multidrug-resistant (MDR) and extended-spectrum beta-lactamase (ESBL) producing *Salmonella enterica* serovar Infantis strains isolated from the broiler food chain production through whole genome sequencing (WGS), PCR, and morphotype association assays. Biofilm formation was quantified by testing the strains at two different temperatures, using 96-well polystyrene plates. The rough and dry colony (rdar) morphotype was assessed visually on Congo red agar (CRA) plates. Based on our results, all tested *S*. Infantis strains produced biofilm at 22 °C with an rdar morphotype, while at 37 °C, all the isolates tested negative, except one positive. Most isolates (58.75%) exhibited strong biofilm production, while 36.25% showed moderate production. Only 5 out of 80 (6.25%) were weak biofilm producers. WGS analysis showed the presence of the *fim* cluster (*fim*ADF) and the *csg* cluster (*csgBAC* and *csgDEFG*), also described in *S*. Typhimurium, which are responsible for fimbriae production. PCR demonstrated the presence of *csgD*, *csgB*, and *fimA* in all 80 *S*. Infantis strains. To our knowledge, this is the first study comparing the effects of two different temperatures on the biofilm formation capacity of ESBL producing *S*. Infantis from the broiler production chain. This study highlights that the initial biofilm components, such as curli and cellulose, are specifically expressed at lower temperatures. It is important to emphasize that within the broiler farm, the environmental temperature ranges between 18–22 °C, which is the optimum temperature for in vitro biofilm formation by *Salmonella* spp. This temperature range facilitates the expression of biofilm-associated genes, contributing to the persistence of *S*. Infantis in the environment. This complicates biosecurity measures and makes disinfection protocols on the farm and in the production chain more difficult, posing serious public health concerns.

## 1. Introduction

*Salmonella* infections pose significant global public health concerns related to non-typhoidal *Salmonella enterica* serotypes [1]. Animal-derived food products, particularly eggs and poultry meat, represent the predominant vehicles for transmission of *Salmonella* infections [2]. In recent years, *Salmonella* surveillance programs, particularly in poultry production (e.g., in the EU and the US), have led to noticeable changes in the trends of food-borne salmonellosis and its associated serotypes [3]. Poultry, especially chickens and turkeys, are commonly infected with *Salmonella*, without showing visible symptoms, leading to subclinical infections or individuals acting as healthy carriers. Since 2010, there has been a substantial increase in the prevalence of *S*. Infantis, which has become the most commonly isolated serovar in broiler production in numerous member states of the United Nations since 2014. Presently, broilers and their byproducts (flocks, meat, and meat products) account for 95% of *S*. Infantis isolates [4]. Significantly, *S*. Infantis has been identified as possessing diverse genetic strategies that augment its epidemiological fitness. These strategies encompass the acquisition and transmission of antimicrobial resistance (AMR), the presence of mobile virulence genes, and the capability to form biofilm [5,6,7].

The increased impact of *S*. Infantis infections is further complicated by occurrence of resistance to the highest priority “critically important antimicrobials” (CIAs), as well the emergence of multidrug-resistant (MDR) strains isolated from food-producing animals and their derivates [8]. These are resistant to penicillins, cephalosporins, and fluoroquinolones, and are often extended-spectrum beta-lactamases (ESBLs) producers. Third- and fourth-generation cephalosporins represent first-choice drugs for the treatment of serious bacterial infections, and the possibility of therapeutic failure in humans is higher during infections caused by bacteria such as *Salmonella* spp., *Klebsiella pneumoniae*, or *Escherichia coli* that produce ESBLs [8].

Despite being an intestinal pathogen, *S*. Infantis can readily persist outside the organism’s host by forming biofilm as an important survival adaptation mechanism. Furthermore, studies have shown that biofilm formation provides tolerance to various stressors (temperature, pH, disinfection, antibiotics, etc.) encountered in both host and non-host environments [9]. For this reason, the eradication of *Salmonella* spp. within even one facility, such as a farm, becomes very difficult. Bacteria within biofilm pose a considerable challenge for elimination compared to planktonic cells, as they are effectively shielded against antibiotics, disinfectants, host immune response, and environmental stress conditions [10]. Consequently, *Salmonella* can persist on abiotic surfaces in farms and food processing plants, progressing through the food chain via food contamination, and ultimately reaching human hosts. Several virulence factors play distinct roles in the pathogenesis of *Salmonella* infections, including flagella, capsules, plasmids, adhesion systems, and type III secretion systems (TTSS) encoded in *Salmonella* pathogenicity islands SPI-1, SPI-2, and others [11]. *Salmonella* spp. requires multiple genes for full virulence, and many of these genes are located in pathogenicity island (PAIs) within the chromosome that are also found to be present in *S*. Infantis. Cellulose and curli fimbriae are the most important components in biofilm formation. These components of the extracellular polymeric substance are relevant because they should be broken up or dissolved during the cleaning process to allow disinfectants to access bacterial cells [12]. Curli is an essential component of the *Salmonella* extracellular matrix, and without it, biofilm formation is greatly reduced. Baugh et al. (2012) showed that transcriptional repression of the two curli biosynthetic operons is responsible for the loss of curli production in the efflux mutants and consequently no biofilm formation is observed [13,14]. Variations in the expression of cellulose and curli fimbriae result in the development of different morphotypes. In vitro, the red, dry, and rough (rdar) morphotype is characterized by the co-expression of cellulose fimbriae and curli. This phenotype is associated with the *csgD* and *adrA* genes. The *csgD* gene is encoded within the curli *csgDEFG* operon, contributing to biofilm production through its important role in synchronizing the expression of several determinants of this process [15]. The purpose of this study was to investigate the biofilm-forming ability of 80 MDR and ESBL-producing *S*. Infantis strains isolated from the broiler food chain production. Moreover, PCR and whole genome sequencing were applied in order to detect genes involved in biofilm production.

## 2. Results

### 2.1. Biofilm Quantification

**Figure 1:**
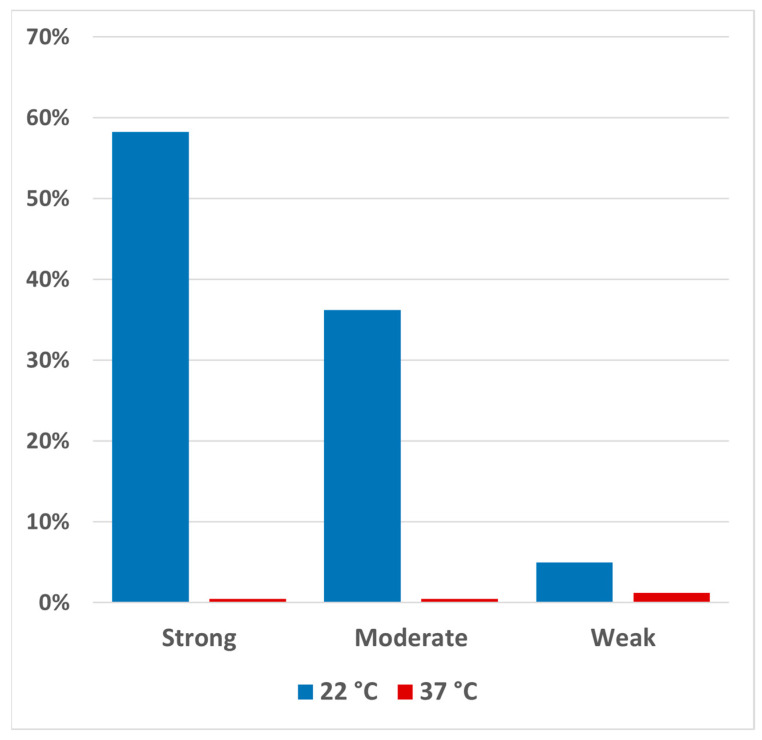
Percentage distribution of the 80 *S.* Infantis isolates assessed in this work in terms of biofilm-producing capacity at 22 and 37 °C.

The results obtained by testing the 80 *S.* Infantis strains at 22 °C and 37 °C are shown in Figure 1. Overall, all isolates were biofilm producers at 22 °C, with varying degrees of production (Table S1; Figure 1). Specifically, 47 out of 80 isolates (58.75%) exhibited strong biofilm production, 29 out of 80 (36.25%) showed moderate production, and only 4 out of 80 (6.25%) were identified as weak biofilm producers. In contrast, 79 isolates tested at 37 °C were non-biofilm producers. Only one isolate was a weak biofilm producer at this temperature (Table S2).

### 2.2. Morphotype Evaluation

**Figure 2:**
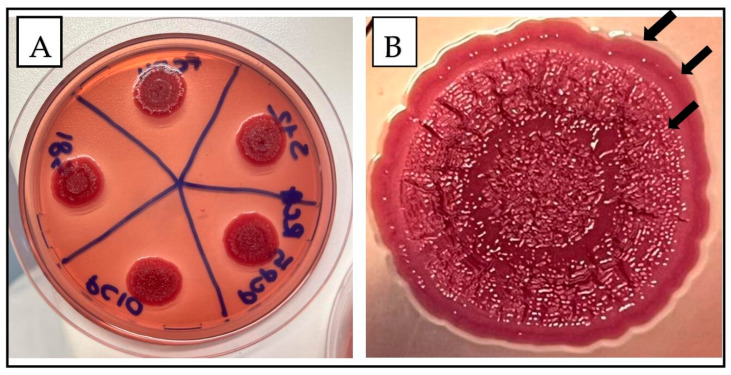
The rdar morphotype of *S*. Infantis isolates. (**A**) represents the morphotype of all the isolates analyzed (80 strains). Conversely, (**B**) illustrates the three distinct zones observed in the rdar morphotype.

The morphological characteristics of the isolates tested by the CRA method are represented in Figure 2. As all isolates produced biofilm at different levels at 22 °C, they all expressed the rdar morphotype (Figure 2A). We observed three distinct regions characteristic of the rdar morphotype (Figure 2B; from top to the bottom): the smooth and white region at the colony’s edge, indicative of no Congo red binding; a narrow, red, and smooth region, suggesting moderate expression of curli and cellulose; and a large central region characterized by a rough surface topology, with elaborate wrinkles and a deep red coloration, indicating high expression levels of curli and cellulose.

### 2.3. Molecular Characterization

The PCR analysis indicated that all 80 *S*. Infantis strains carried the *csgD*, *csgB*, and *fimA* genes. Subsequent WGS analysis of the four sequenced strains, consisting of three strong biofilm producers and one moderate biofilm producer, showed the following results. Three strong biofilm producers presented the *fim (ADF)* gene cluster, and one of them also expressed the CSG cluster (*csgA*, *csgB*, *csgC*, *csgD*, *csgE*, *csgF*, *csgG*). The moderate biofilm producer sequenced carried only the *fimA1* gene.

## 3. Discussion

The ability of microbes to resist the action of antimicrobial substances has become a serious global concern due to the rapid transmission of *S*. Infantis and its impact on human, animal, and environmental health. Drug resistance associated with biofilms is a highly challenging phenomenon [16]. Their ability to provide a niche for tolerant and persistent cells, together with the barrier properties of the biofilm matrix, is the main obstacle to eradicating biofilms in different livestock farm areas, complicating internal biosecurity measures and leading to chronic subclinical infections and economic losses [17]. Moreover, they contribute significantly to food cross-contamination, impacting the entire food production chain [18]. *S*. Infantis is recognized as an emerging serotype at the European level, exhibiting widespread prevalence within the poultry industry. Currently, it stands as one of the most frequently isolated serotypes, not only in broiler farms and poultry meat, but also in turkey farms and their related products [19,20]. According to a recent meta-analysis, MDR *S.* Infantis isolates are most frequently reported from broiler meat (68.3%), in particular from grilled chicken, liver, and drumsticks, and thighs [21,22].

To our knowledge, this is the second study conducted on *S*. Infantis strains isolated along the broiler food production chain and the first one conducted in Italy. However, the first work conducted considered only *S*. Infantis MDR, but not the ESBLs strains [23]. In the present study, we tested the biofilm-production capacity at 22 °C and 37 °C, with the aim of investigating how environmental conditions influence biofilm production in *S*. Infantis. A temperature of 22 °C is commonly found in broiler farms following the weaning period. Therefore, we selected this temperature to replicate the environmental conditions of the farm, and in parallel, we also assessed a temperature of 37 °C, the growth temperature of Enterobacteriaceae in their homeothermic vertebrate hosts. We observed that *S*. Infantis strains were capable of producing biofilms when tested at 22 °C, but did not produce biofilms when tested at 37 °C. Most of the studies reported the biofilm formation ability of *S*. Derby, *S*. Mbandaka, and *S*. Enteritidis. In particular, Lamas et al. (2016) evaluated the biofilm production of 67 *Salmonella* strains isolated from poultry farms [24]. The study was carried out at four different temperatures (6 °C, 20 °C, 28 °C, and 37 °C) and in low or high nutrient concentrations. The results showed that *S. enterica* subsp. *arizonae* and *S*. Typhimurium had a greater ability to form biofilms at 20 °C. Other studies have also confirmed that the range of biofilm production for *S*. Typhimurium is below 25 °C [25,26]. Our research findings on *S*. Infantis are consistent with those of the studies conducted on other *Salmonella enterica* serovars, as all the tested strains were biofilm producers at 22 °C. It is important to emphasize the relationship between biofilm formation and environmental conditions in the broiler farm, in which a temperature between 18 °C and 22 °C is optimal for biofilm formation by *S*. Infantis. This result suggests that *S.* Infantis implemented biofilm production as a survival strategy under stress conditions, such as low temperature and low nutrient content. Other authors reported that *S*. *enterica* subsp. *enterica* can also persist within biofilms in food environments, thereby facilitating food cross-contamination and rendering it unsuitable for consumption [27]. On the other hand, this notable difference may also be attributed to enhanced cell adhesion at a lower temperature [28]. Nguyen et al. (2014) showed that the rate of biofilm formation increased with increasing temperature and pH, while the number of attached cells after 240 h decreased with increasing temperature. This result could be due to the production of the thin aggregative fimbriae in *S*. Infantis strains in a temperature-dependent environment [29,30].

In the present study, all tested *S*. Infantis strains carried *csgD*, which is responsible for the production of cellulose and the expression of the rdar morphotype, *csgB*, coding the major subunit of curli, and *fimA*, coding the major fimbriae subunit. These genes can lead to a morphotype characterized by the expression of the adhesive extracellular matrix components cellulose and curli fimbriae [31]. The *csgD* gene is a component of the *csgBAC-csgDEFG* complex and plays a crucial role as a primary transcriptional regulator in controlling *Salmonella* biofilm formation. The curli fimbriae are involved in the biofilm formation process as essential structures for initiating adhesion to surfaces. The fimbriae gene cluster (*fim*) is composed of an operon that encodes structural and regulatory components. Bacterial adherence plays a critical role in the initial phase of infection. Fimbriae, present on the surface of bacteria, aid in their attachment to specific receptors on host cells or surfaces. These receptors are located in various tissues, including the urinary tract, intestinal mucus, and epithelial cells [32,33].

Four out of 80 strains were weak biofilm producers, although they exhibited an rdar morphotype and were *csgD*, *adrA*, and *fimA* positive. This may indicate that the process of biofilm formation in *S*. Infantis could be influenced by other genes that were not assessed here. According to several studies, under stress conditions, the production of curli and cellulose is likely to increase [34,35]. *CsgD*, a crucial regulator of biofilm formation in *Salmonella*, controls the expression of the main biofilm components and facilitates the transitions between different behavioral modes. However, *csgD* typically exhibits a low basal transcription level, and its expression is strongly influenced by various environmental factors, such as temperature, oxygen levels, nutrients, osmolarity, ethanol, iron, and pH. Furthermore, the comparison of the rdar phenotype with other biofilm analysis systems can be difficult [16]. As an additional consideration, our results concerning the weak biofilm formers could be derived from the application of two different methodologies. Studies demonstrated that 30% of the functional genome of *S*. Typhimurium was differentially regulated between agar and broth culturing. This further complicates the comparison between the rdar phenotype and other biofilm testing systems, highlighting the need for careful interpretation and consideration of the experimental conditions [36].

Our data showed that all the strains assessed in this study, previously identified as MDR and ESBL positive, exhibited biofilm-producing capabilities at varying degrees. These results suggest a positive correlation between the capacity to form biofilms and MDR mechanisms, in agreement with the results of other previous studies [37,38]. The ability to form biofilms can enhance antibiotic tolerance and confer an adaptive resistance to antibiotics. Narasanna et al. (2017) showed that cefetoxime at a sub-inhibitory (sub-MIC) level efficiently induces biofilm formation and promotes changes in the morphology of the cell, impacting the ultrastructure and antigenicity of the microorganisms, as well as their adherence to epithelial cells [39]. Another probable reason could be the presence of MDR efflux pumps or MDR operons that function in both antibiotic resistance and biofilm formation [40].

However, the development of in situ disinfection protocols (DP) for livestock farming requires an understanding of the optimal concentrations and exposure times for an efficient management of pathogens within biofilms; this is crucial for mitigating antimicrobial resistance and addressing biofilm-related issues throughout the food production chain, from farm to fork. Clinical and industrial practices must conform to standards to ensure consistency, scalability, reproducibility, and cost-effectiveness, since that DP, even at their recommended concentrations, may not fully deactivate target microorganisms within biofilms, whether single or multi-species [4].

## 4. Materials and Methods

### 4.1. Collection and Isolate Identification

This study investigated 80 *S*. Infantis strains selected for ESBLs and the phenotypic antibiotic resistance profile. The strains were isolated in Italy from 2016 to 2017 along the broiler production chain (farms and slaughterhouses), following standard ISO procedures (ISO 6579-1:2017/Amd 1:2020) [41]. Detailed information for sample collection and *Salmonella* Infantis isolation and identification were described in a previous study [41]. Briefly, pre-enrichment of all samples was performed in buffered peptone water (BPW) at 37 °C overnight, and selective enrichment was then performed on the pre-enriched cultures by inoculation on Rappaport-Vassiliadis (RV) agar at 41.5 °C for 24 h. Finally, a loopful of material from the RV agar was transferred to xylose-lysine-deoxycholate (XLD) agar and Chromogenic Salmonella Agar Base and incubated at 37 °C for 24 h. The confirmed isolates were serotyped by direct slide agglutination with the specific antisera (Statens Serum Institute, Copenhagen, Denmark), according to the Kaufmann–White–Le Minor scheme [42]. The bacterial strains were stored in Tryptic Soy Broth (TSB), with the addition of 15% (*v*/*v*) glycerol at -80 °C, until use. The bacterial cultures were then inoculated in Luria Bertani (LB) broth and incubated at 37 °C for 18 h, with orbital shaking (200 rpm).

### 4.2. Bacterial Cell Adhesion Analysis

The ability to adhere and form biofilm was determined according to the methods of Stepanovic et al. (2007) with the modifications described below. Briefly, the bacterial strains were processed in quadruplicate in three independent experiments, using freshly prepared reagents and media [43,44]. Before each experiment, the bacterial cultures were streaked and incubated overnight in Mueller Hinton Agar at 37 ± 1 °C to assess their purity. The bacterial cultures were then transferred into LB broth (tryptone 10 g/L, yeast extract 5 g/L) at low concentrations of NaCl (<0.5%) (Liofilchem, Roseto degli Abruzzi, TE, Italy).

#### 4.2.1. Microtiter Plate Assessment

Biofilm formation was carried out in sterile lidded flat-bottomed 96-well treated polystyrene microtiter plates for tissue culture (BD Falcon, Turin, Italy). Then, 100 µL of standardized bacterial suspension (1 × 10^5^ CFU/mL) was aliquoted into a 96-well flat-bottomed microplate and incubated for 48 h at 23 ± 1 °C and 37 °C [23]. A negative control, represented by LB broth only, and a positive control, represented by a strong biofilm-producing strain (*Salmonella* Typhimurium ATCC 14028), were used for validation and confirmation purposes. After incubation, the liquid was discarded, and each well was gently washed three times with 300 µL of sterile PBS (pH 7.2) (Sigma, St. Louis, MI, USA), taking care to preserve the integrity of any biofilm that may have been formed. The plates were then incubated at 60 °C for 60 min to allow them to dry. A standard amount (150 µL) of crystal violet (0.5%) (Sigma, St. Louis, MI, USA) was added, and the mixture was held for 15 min at room temperature. The excess crystalloid was aspirated, the sample was washed twice in water, and then the microplate was dried for 30 min at 37 °C. At this point, the cells were resolubilized in 150 µL of 96% ethanol, covered to prevent evaporation, and left at room temperature for 30 min before reading. The optical density of each well was measured at 570 nm (OD570), using a microplate reader (Tecan group Ltd., Männedorf, Switzerland).

#### 4.2.2. Biofilm Quantification Analysis

According to Stepanovic, the cut-off value distinguishing the biofilm-producing from the non-biofilm-producing strains was defined for the interpretation of the results obtained [44]. For the analysis of biofilm production, we determined the cut-off OD value (ODc), which is three standard deviations (SD) above the OD of the negative control (ODc = the mean OD570 of the negative control + 3 SD of the negative control). The final OD value of a tested strain is expressed as the average OD value of the strain reduced by the ODc value. This method allowed us to categorize the bacteria, based on the previously determined OD values, into the following categories: non-biofilm producers (OD570 ≤ ODc); weak biofilm producers (ODc ≤ OD570 ≤ 2× ODc); moderate biofilm producers (2× ODc ≤ OD570 ≤ 4× ODc); and strong biofilm producers (OD570 > 4× ODc) [44].

### 4.3. S. Infantis Morphotype (Colony Morphology and Cellulose Production)

All *Salmonella* strains were assessed on Congo red agar (CRA) plates, based on morphological colony characteristics, to determine the biofilm morphotype of each strain. The morphotype monitoring assay was performed according to the method of Romling and Rohde (1999), with minor adaptations [15]. Briefly, 5 µL of overnight cultures were dropped into LB agar, without NaCl (yeast extract 5 g/L, bacto-tryptone 10 g/L, agar 15 g/L), supplemented with 40 µg/mL Congo red (Sigma-Aldrich, St. Louis, MI, USA), and 20 µg/mL Coomassie Brillant Blue (Sigma-Aldrich), and incubated at 22 °C for 96 h. The morphotypes were classified into four groups: rdar (red, dry, and rough)—indicating curli fimbriae and cellulose; (ii) bdar (brown, dry, and rough)—indicating only curli fimbriae; (iii) pdar (pink, dry, and rough)—indicating only cellulose; and iv) saw (smooth and wet), indicating neither cellulose nor fimbriae. All assays were performed in triplicate [16,45].

### 4.4. Molecular Characterization

#### 4.4.1. Detection of Biofilm Genes

Genomic DNA was extracted from individual colonies using the GenElute Bacterial Genomic DNA Kit (Sigma-Aldrich, Darmstadt, Germany), according to the manufacturer’s protocol, using overnight cultures in TSB. Extracted DNA quantity and quality were determined by spectrophotometry (NanoDrop, Thermo Fisher Scientific, Milan, Italy) and electrophoresis on 1% agarose gel, respectively. All isolates were screened by a single PCR assay targeting the *fimA*, *csgB*, and *csgD* genes as mediators of cell adhesion in the first step of biofilm production, as previously described [46,47,48] (Table 1). PCRs were carried out in a 25 μL PCR mixture containing 0.5 μM of each primer. *S. aureus* ATCC 29213 was employed as a negative control for the three genes tested.

#### 4.4.2. Whole Genome Sequencing

The genomic DNA of four *S*. Infantis isolates (three strong and one moderate biofilm producer) was used for library preparation employing a commercial kit (Nextera XT, Illumina San Diego, CA, USA). The isolates analyzed in the present study derive from a previous work in which they were sequenced and investigated for antibiotic-resistance genes [7]. The libraries were sequenced using paired-end Illumina MiSeq, and the quality of the raw reads was checked using FastQC (http://www.bioinformatics.babraham.ac.uk/projects/fastqc/, accessed on 15 January 2024), removing those showing low quality (Phred score < 20) and fewer than 70 nucleotides. Processed reads (coverage 100×) were de novo assembled using SPAdes, version 3.14. [49], and the generated contigs were passed to CSAR v. 1.1.1 to build the scaffolds with more than 200 nucleotides in length. Subsequently, the genome assembly quality check was performed with QUAST v. 4.3 [50] and the sequences were annotated using Prokka v. 1.12 [51]. The genome sequences were analyzed for the presence of genes related to biofilm production.

## 5. Conclusions

The presence of bacterial biofilms in food processing environments represents a significant public health concern, as bacteria within biofilms are better protected from various external stresses compared to their planktonic counterparts. This highlights the direct link between food production chain contamination and bacterial biofilms. Our study contributes to the current knowledge regarding the biofilm-forming capacity of MDR and ESBL *S*. Infantis at the temperatures found in the poultry meat production chain. It also represents the first evaluation of biofilm production conducted in Italy along the broiler production chain. Disinfection protocols and internal biosecurity in broiler farms and slaughterhouses might be severely hampered by the *S*. Infantis biofilm-forming ability and enhanced by stress and environmental conditions, as the expression of curli and cellulose is strongly dependent on temperature, thus leading to an increase in the persistence of this bacterium in the environment.

## Figures and Tables

**Table 1:** Target genes encoding the cell attachment mechanisms and respective primer sequences.

Target Genes	Sequence of Primers (5′–3′)	Product Length	References
*csgD*	F: TCCTGGTCTTCAGTAGCGTAA R: TATGATGGAAGCGGATAAGAA	168 bp	[48]
*csgB*	F: ATCAGGCGGCCATTATTGGTCAAG R: TGCTGTTTTCTGCGTACCGTACTG	275 bp	[47]
*fimA*	F: TGCCTTTCTCCATCGTCC R: TGCGGTAGTGCTATTGTCC	134 bp	[46]

## Data Availability

Data are contained within the article and Supplementary Materials.

## Supplementary Materials

The following supporting information can be downloaded at: https://www.mdpi.com/article/10.3390/antibiotics13070595/s1, Table S1. Comprehensive analysis of biofilm production levels in three independent experiments for the 80 tested Salmonella isolates at 22 °C. Table S2. Comprehensive analysis of biofilm production levels in three independent experiments for the 80 tested Salmonella isolates at 37 °C.
