# Supplementary material for: High Biofilm-Forming Multidrug-Resistant Salmonella Infantis Strains from the Poultry Production Chain
Source: Antibiotics (Basel). 2024 Jun 27;13(7):595. doi: 10.3390/antibiotics13070595 (PMC11273867; doi:10.3390/antibiotics13070595)
Supplement: Supplementary file 1 [file antibiotics-13-00595-s001.zip › antibiotics-3063142-supplementary.pdf]

Table S1. Comprehensive analysis of biofilm production levels in three independent experiments for the 80 tested Salmonella isolates at 22˚C.

Sample	Origin	Expt 1	Expt 2	Expt 3	Standard deviation	OD Mean	Biofilm production degree
S21	Slaughter	1.5266	1.8881	1.8794	0.21	1.26	Moderate
S18	Slaughter	1.3785	1.8284	1.5528	0.23	1.08	Moderate
18S	Slaughter	1.2935	1.7646	1.4743	0.24	1.00	Weak
1S	Slaughter	1.458	2.1751	1.8604	0.36	1.32	Moderate
PC10	Slaughter	1.6473	2.4844	1.9333	0.43	1.51	Moderate
PC5	Slaughter	1.4015	2.0102	1.562	0.32	1.15	Moderate
PC11	Slaughter	1.4715	2.3982	1.8313	0.47	1.39	Moderate
PCP3	Slaughter	1.5811	1.9913	1.515	0.26	1.19	Moderate
S9	Slaughter	1.7956	1.3814	1.7356	0.22	1.13	Moderate
PCP1	Slaughter	1.819	2.14	2.0483	0.17	1.50	Moderate
19S	Slaughter	1.38	1.6747	1.7676	0.20	1.10	Moderate
3S	Slaughter	1.7692	1.9431	2.2323	0.23	1.47	Moderate
R3	Slaughter	1.6292	1.9161	1.9319	0.17	1.32	Moderate
PC12	Slaughter	1.6285	2.1471	2.374	0.38	1.54	Moderate
S30	Farm	1.3256	1.8758	1.8688	0.32	1.18	Moderate
S22	Slaughter	1.53	1.4571	1.4596	0.04	0.97	Weak
PC6	Slaughter	1.6578	1.4493	1.8422	0.20	1.14	Moderate
PCP5	Slaughter	1.6929	1.8417	2.4599	0.41	1.49	Moderate
R1	Slaughter	1.6682	1.7894	2.3463	0.36	1.43	Moderate
PCP4	Slaughter	1.7499	1.8527	2.5718	0.45	1.55	Moderate
R2	Slaughter	1.5086	1.3433	2.2483	0.48	1.19	Moderate
22S	Slaughter	1.445125	1.4003	1.61635	0.11	1.17	Moderate
20S	Slaughter	1.709	1.47175	2.00755	0.27	1.41	Strong
R4	Slaughter	1.4758	1.5427	2.8332	0.77	1.63	Strong
16S	Slaughter	1.2361	1.49935	1.9195	0.34	1.24	Moderate
12S	Slaughter	0.6068	0.776775	0.82175	0.11	0.42	Weak
21S	Slaughter	1.389	1.9131	2.08385	0.36	1.48	Strong
7S	Slaughter	1.40095	1.85455	2.0778	0.34	1.46	Strong
LETTB TII	Farm	1.7797	1.83685	1.86685	0.04	1.51	Strong
E	Farm	1.2728	1.801475	2.107025	0.42	1.41	Strong
III F5	Farm	1.767775	1.980075	3.0037	0.66	1.93	Strong
II P2	Farm	1.7443	1.510025	2.83385	0.71	1.71	Strong
I P9	Slaughter	1.582825	2.169325	2.730875	0.57	1.85	Strong
A	Farm	2.07675	2.161375	2.573125	0.27	1.95	Strong
S13	Slaughter	1.89855	2.6588	2.398475	0.39	2.00	Strong
TC5B	Farm	1.981875	2.195075	2.52925	0.28	1.92	Strong
10S	Farm	1.942725	1.91935	2.376825	0.26	1.76	Strong
S7	Slaughter	1.870275	2.044	1.840125	0.11	1.60	Strong
PC2	Slaughter	1.683225	2.331225	2.3759	0.39	1.81	Strong
PC3	Slaughter	1.6449	2.19125	2.273325	0.34	1.72	Strong
PC1	Slaughter	1.838825	2.283575	2.322725	0.27	1.83	Strong
S17	Slaughter	1.61145	2.121775	2.325025	0.37	1.70	Strong

R5	Slaughter	1.659575	1.508275	2.1351	0.33	1.41	Strong
4S	Slaughter	1.8054	1.729775	2.164375	0.23	1.54	Strong
13S	Slaughter	2.04905	1.59645	2.14905	0.29	1.57	Strong
5S	Slaughter	2.109475	1.71675	2.2342	0.27	1.66	Strong
S2	Farm	1.791825	1.73495	2.0184	0.15	1.49	Strong
PC13	Slaughter	2.1274	2.267025	2.746	0.32	2.02	Strong
11S	Farm	1.444	1.070475	1.6799	0.31	1.04	Moderate
9S	Farm	1.704075	2.22495	2.276125	0.32	1.71	Strong
IIF4	Slaughter	1.6841	1.469925	1.90115	0.22	1.33	Moderate
O	Slaughter	0.86085	0.8004	0.797675	0.04	0.46	Weak
S31	Slaughter	2.018225	1.813	2.17435	0.18	1.64	Strong
IIP10	Slaughter	2.0293	1.8513	2.5026	0.34	1.77	Strong
IIIF2	Slaughter	2.081725	1.911175	2.385175	0.24	1.77	Strong
IIF2	Slaughter	2.50995	2.0145	2.475525	0.28	1.98	Strong
S15	Slaughter	2.45725	2.1483	2.372225	0.16	1.97	Strong
S6	Farm	2.25925	2.558975	2.76805	0.26	2.17	Strong
F	Farm	1.439225	1.5414	1.304275	0.12	1.10	Moderate
S8	Slaughter	1.495575	1.69285	1.800375	0.15	1.34	Strong
IIIF1	Farm	1.359925	1.208825	1.454025	0.12	1.02	Moderate
IIP7	Slaughter	1.459225	1.6233	1.2727	0.18	1.13	Moderate
S4	Farm	2.207775	2.31405	1.544925	0.42	1.70	Strong
17S	Slaughter	2.025775	1.841275	1.01825	0.54	1.30	Strong
S24	Slaughter	1.6723	1.8462	1.098525	0.39	1.21	Moderate
S25	Farm	2.01435	1.7543	1.67315	0.18	1.49	Strong
C	Slaughter	1.97205	1.75425	1.7099	0.14	1.49	Strong
S16	Slaughter	1.7293	1.536025	1.810125	0.14	1.37	Strong
8S	Slaughter	1.92365	2.050775	1.93525	0.07	1.64	Strong
R6	Slaughter	2.430475	2.3147	2.029825	0.21	1.93	Strong
IP7	Farm	1.493325	1.236325	1.510325	0.15	1.10	Moderate
S19	Slaughter	1.950275	1.16635	1.71765	0.40	1.30	Strong
PCP2	Slaughter	1.838875	1.491375	2.084475	0.30	1.49	Strong
PC9	Slaughter	2.060675	1.610025	2.2124	0.31	1.65	Strong
PC4	Slaughter	1.6357	1.3045	2.039925	0.37	1.34	Strong
PC8	Slaughter	2.6202	2.21985	2.298825	0.21	2.06	Strong
IP1	Farrm	2.009225	1.4344	2.033625	0.34	1.51	Strong
S29	Slaughter	1.9149	1.163325	1.6191	0.38	1.25	Moderate
IP8	Farm	1.913775	1.224075	2.179425	0.49	1.46	Strong
S12	Farm	1.3785	1.8284	1.5528	0.23	1.28	Strong
Negative Control		0.430425	0.4002	0.398838	0.02	0.23	Negative
Positive Control		2.2288	2.753713	2.876125	0.34	1.47	Positive (strong)

Table S2. Comprehensive analysis of biofilm production levels in three independent experiments for the 80 tested Salmonella isolates at 37˚C.

Sample	Origin	Expt 1	Expt 2	Expt 3	Standard deviation	OD Mean	Biofilm production degree
S21	Slaughter	0.0378	0.0905	0.0873	0.03	-0.01	Negative
S18	Slaughter	0.1535	0.1205	0.0820	0.04	0.036799	Negative
18S	Slaughter	0.0843	0.0920	0.0765	0.01	0.002382	Negative
1S	Slaughter	0.1900	0.1068	0.0778	0.06	0.042966	Negative
PC10	Slaughter	0.1113	0.1058	0.1158	0.01	0.029049	Negative
PC5	Slaughter	0.0740	0.0783	0.0683	0.01	-0.00837	Negative
PC11	Slaughter	0.0745	0.0723	0.1194	0.03	0.006849	Negative
PCP3	Slaughter	0.0883	0.0878	0.0988	0.01	0.009716	Negative
S9	Slaughter	0.1048	0.0805	0.0673	0.02	0.002299	Negative
PCP1	Slaughter	0.0993	0.2423	0.0903	0.09	0.062049	Negative
19S	Slaughter	0.0702	0.0625	0.1613	0.05	0.016116	Negative
3S	Slaughter	0.0382	0.0555	0.1975	0.09	0.015199	Negative
R3	Slaughter	0.1112	0.0915	0.0725	0.02	0.009866	Negative
PC12	Slaughter	0.1027	0.0983	0.0722	0.02	0.009182	Negative
S30	Farm	0.0892	0.0948	0.1695	0.04	0.035949	Negative
S22	Slaughter	0.1027	0.0965	0.1068	0.01	0.020116	Negative
PC6	Slaughter	0.1145	0.0825	0.0840	0.02	0.011799	Negative
PCP5	Slaughter	0.0951	0.1442	0.0962	0.03	0.029972	Negative
R1	Slaughter	0.0633	0.0632	0.1362	0.04	0.005699	Negative
PCP4	Slaughter	0.1274	0.1332	0.1002	0.02	0.038399	Negative
R2	Slaughter	0.1220	0.0792	0.1032	0.02	0.019599	Negative
22S	Slaughter	0.1148	0.0780	0.0958	0.02	0.014299	Negative
20S	Slaughter	0.1203	0.0895	0.1050	0.02	0.023049	Negative
R4	Slaughter	0.1130	0.0930	0.1000	0.01	0.020132	Negative
16S	Slaughter	0.1112	0.0790	0.0977	0.02	0.014099	Negative
12S	Slaughter	0.1167	0.0822	0.1012	0.02	0.018166	Negative
21S	Slaughter	0.1270	0.0928	0.1235	0.02	0.032549	Negative
7S	Slaughter	0.1413	0.0925	0.1145	0.02	0.034216	Negative
LETTB TII	Farm	0.1125	0.0850	0.0940	0.01	0.015299	Negative
E	Farm	0.1092	0.0852	0.0937	0.01	0.014166	Negative
III F5	Farm	0.0943	0.1023	0.0724	0.02	0.007792	Negative
II P2	Farm	0.1407	0.1023	0.0992	0.02	0.032199	Negative
I P9	Slaughter	0.0994	0.0897	0.0589	0.02	0.000799	Negative
A	Farm	0.1204	0.0995	0.0893	0.02	0.021202	Negative
S13	Slaughter	0.0432	0.1277	0.1598	0.06	0.028366	Negative
TC5B	Farm	0.0888	0.0675	0.0565	0.02	-0.01093	Negative
10S	Farm	0.0984	0.0677	0.0979	0.02	0.006132	Negative
S7	Slaughter	0.0798	0.1679	0.1712	0.05	0.057766	Negative
PC2	Slaughter	0.2575	0.1923	0.2094	0.03	0.137832	Positive (weak)
PC3	Slaughter	0.0996	0.0349	0.1698	0.07	0.019566	Negative
PC1	Slaughter	0.0348	0.1649	0.0932	0.07	0.015766	Negative
S17	Slaughter	0.1023	0.1765	0.0931	0.05	0.042099	Negative

R5	Slaughter	0.0895	0.0691	0.1280	0.03	0.013666	Negative
4S	Slaughter	0.0731	0.0967	0.1014	0.02	0.008532	Negative
13S	Slaughter	0.0920	0.0593	0.1034	0.02	0.003032	Negative
5S	Slaughter	0.1068	0.0923	0.0694	0.02	0.007616	Negative
S2	Farm	0.1034	0.0947	0.0639	0.02	0.005466	Negative
PC13	Slaughter	0.0783	0.0985	0.1034	0.01	0.011516	Negative
11S	Farm	0.0723	0.0596	0.1338	0.04	0.006682	Negative
9S	Farm	0.0878	0.0793	0.1038	0.01	0.008416	Negative
IIF4	Slaughter	0.0805	0.1039	0.1193	0.02	0.019366	Negative
O	Slaughter	0.0994	0.0785	0.0938	0.01	0.008699	Negative
S31	Slaughter	0.1206	0.0993	0.0973	0.01	0.023866	Negative
IIP10	Slaughter	0.1039	0.0736	0.1495	0.04	0.027132	Negative
IIIF2	Slaughter	0.1034	0.1107	0.0876	0.01	0.018699	Negative
IIF2	Slaughter	0.0594	0.0596	0.1394	0.05	0.004266	Negative
S15	Slaughter	0.1103	0.0794	0.0883	0.02	0.010799	Negative
S6	Farm	0.0794	0.0847	0.0938	0.01	0.004099	Negative
F	Farm	0.1849	0.0938	0.0529	0.07	0.028666	Negative
S8	Slaughter	0.0695	0.0958	0.0847	0.01	0.001466	Negative
IIIF1	Farm	0.0736	0.0938	0.1694	0.05	0.030399	Negative
IIP7	Slaughter	0.1485	0.0847	0.1038	0.03	0.030466	Negative
S4	Farm	0.0384	0.1347	0.1039	0.05	0.010466	Negative
17S	Slaughter	0.0849	0.0695	0.0929	0.01	0.000559	Negative
S24	Slaughter	0.1039	0.1104	0.0603	0.03	0.009666	Negative
S25	Farm	0.0799	0.0948	0.0723	0.01	0.000466	Negative
C	Slaughter	0.1039	0.0938	0.0794	0.01	0.010499	Negative
S16	Slaughter	0.0948	0.0894	0.1049	0.01	0.014499	Negative
8S	Slaughter	0.0795	0.0918	0.0796	0.01	0.001759	Negative
R6	Slaughter	0.0968	0.1165	0.1004	0.01	0.022699	Negative
IP7	Farm	0.0958	0.1195	0.0725	0.02	0.014066	Negative
S19	Slaughter	0.0495	0.1194	0.0948	0.04	0.006032	Negative
PCP2	Slaughter	0.0948	0.0694	0.1193	0.02	0.012632	Negative
PC9	Slaughter	0.0847	0.0694	0.1075	0.02	0.005332	Negative
PC4	Slaughter	0.0958	0.1295	0.1094	0.02	0.029699	Negative
PC8	Slaughter	0.1304	0.0739	0.1093	0.03	0.022666	Negative
IP1	Farm	0.0993	0.1406	0.0994	0.02	0.031232	Negative
S29	Slaughter	0.1708	0.0171	0.0918	0.08	0.011362	Negative
IP8	Farm	0.0609	0.1039	0.1116	0.03	0.010266	Negative
S12	Farm	0.0948	0.1374	0.1029	0.02	0.029832	Negative
Negative control		0.0705	0.0590	0.0613	0.01	-0.01828	Negative
Positive control		0.8335	0.6045	0.81525	0.13	0.75108	Strong
